# Temporal dynamics of soil microbial symbioses in the root zone of wolfberry: deciphering the effects of biotic and abiotic factors on bacterial and fungal ecological networks

**DOI:** 10.3389/fpls.2025.1518439

**Published:** 2025-03-10

**Authors:** Mengyuan He, Qianqian Wang, Yiming Wang, Junhua Zhang

**Affiliations:** ^1^ School of Life Sciences, Ningxia University, Yinchuan, China; ^2^ School of Ecology and Environment, Ningxia University, Yinchuan, China; ^3^ State Key Laboratory of Soil and Sustainable Agriculture, Institute of Soil Science, Chinese Academy of Sciences, Nanjing, China

**Keywords:** *Lycium barbarum*, stand age, soil microorganism, ecological network, assembly mechanisms

## Abstract

Long-term monoculture of *Lycium barbarum* significantly affects its productivity and soil health. Soil microbiota, which mediate the sustainable development of soil ecosystems, are influenced by the age of wolfberry plants. However, the comprehensive effects of long-term cultivation of *L. barbarum* on the soil microbial community are not yet fully understood. Here, we assessed the effects of stand age on the diversity, composition, assembly, and symbiotic networks of bacterial and fungal communities in the root zone soil of *L. barbarum* using high-throughput sequencing technology. The results showed that stand age significantly affected the α-diversity of bacterial and fungal communities, as evidenced by the tendency of their Shannon and Chao1 indices to increase and then decrease. At the same time, the structure of soil bacterial and fungal communities was significantly influenced by tree age. However, Proteobacteria (28.77%–32.81%) was always the most dominant bacterial phylum, and Ascomycetes (49.72%–55.82%) was always the most dominant fungal phylum. A number of genus-level biomarkers were also identified in soils associated with roots of trees of varying ages. Additionally, stochastic processes dominated the assembly of soil bacterial communities, whereas the balance between stochastic and deterministic processes in the assembly of fungal communities fluctuated with stand age. The complexity and stability of bacterial and fungal community networks were notably affected by tree age, particularly in networks from 10- and 15-year-old trees. The partial least squares path modeling (PLS-PM) analysis emphasized that stand age can indirectly regulate the diversity and network complexity of both bacterial and fungal communities by influencing soil physicochemical properties. Furthermore, the bacterial community, but not the fungal community, exhibited direct and strong regulation of network complexity. The study offers valuable data for improving the soil quality and fruit yield of *L. barbarum* under long-term continuous cropping, which has implications for the sustainable development of the *L. barbarum* industry.

## 1 Introduction

Wolfberry (*Lycium barbarum*) is a deciduous shrub belonging to the Solanaceae family, primarily found in China and other parts of Asia (Liu et al., 2022). It demonstrates remarkable tolerance to various abiotic stresses, including high salinity, cold, and drought (Zhao et al., 2015). Due to its medicinal and nutritional values, the cultivation of wolfberry has emerged as a key agro-industry contributing to regional economic growth (Li et al., 2023). However, continuous cropping for more than a decade tends to decrease the fruit yield and quality of *L. barbarum*, posing a significant challenge not only to sustainable wolfberry cultivation and economic development but also to the sustainability of the soil ecosystem (Zhang et al., 2018). This decline is primarily attributed to the deterioration of soil quality resulting from inadequate soil management practices, continuous pesticide application, and long-term monocropping systems (Zhu et al., 2022).

Microorganisms are an essential component of the soil ecosystem and play an active role in maintaining plant and soil health (Zhang et al., 2024c). Increases in the years of *L. barbarum* cultivation may have significant effects on the diversity and function of soil microbial communities (Zhang et al., 2023a; He et al., 2024b). For instance, long-term continuous cropping can alter soil physicochemical properties, potentially leading to a reduction in overall microbial community diversity (Song et al., 2024). Continuous cultivation may also result in significant alterations in the microbial community composition (Na et al., 2016; Zhang et al., 2018). Na et al. (2019) explored the relationship between soil microbial communities and the stand age of *L. barbarum*, discovering that long-term monocropping of *L. barbarum* fosters the enrichment of key phytopathogenic fungal genera, such as *Gibberella*, *Alternaria*, and *Stemphylium*. These fungi potentially pose threats to the health of the older *L. barbarum* plants. A recent study further revealed that changes in bacterial community composition, influenced by stand age, can enhance the enrichment of *Fusarium* spp., thereby jeopardizing the soil health of *L. barbarum* fields (Na et al., 2021). Despite the increasing recognition of soil microbial community structure in *L. barbarum* fields, the mechanisms underlying the generation and maintenance of dynamic changes in soil microbial communities due to tree age remain inadequately understood and discussed.

The effect of stand age on soil microbial communities is not only in terms of diversity and community structure, but also in terms of microbial community assembly and interactions (Wang et al., 2022; Zhang et al., 2023b). The process of microbial community assembly is widely recognized in the field of microbial ecology (Nemergut et al., 2013). As previously described, stochastic and deterministic processes, which emphasize “born free” and “environmental filtering”, respectively, may be facilitated or constrained by environmental changes across time and space, thereby influencing microbial community assembly processes (Fargione et al., 2003; Zhou and Ning, 2017). Currently, the assembly of microbial communities has been studied primarily in cash crops such as corn, soybean, wheat, and rice (Liu et al., 2020a; Cao et al., 2024; Liu et al., 2024). How continuous cultivation affects the assembly of soil microbial communities in the root zone of *L. barbarum*, i.e., the balance between stochastic and deterministic processes, is not known. Furthermore, microbial network analysis has been utilized as an important tool to study microbial interactions. Although it does not fully simulate microbial interactions in real environments, it can aid in understanding microbial community responses to external environmental changes (Wang et al., 2023). The complexity and stability of soil microbial networks are typically influenced by a variety of factors (Fierer, 2017). Regarding the effect of *L. barbarum* stand age on microbial networks, only in the study by Na et al. (2021) was it observed that the complexity of young stand networks was higher than that of old stand networks. Although this study spanned multiple tree ages, it only compared networks from younger and older stands and lacked research on smaller scales, specifically comparisons between multiple networks of different tree ages. It is critical to address this, as it is essential for exploring the dynamics of the soil microbiota in *L. barbarum* fields and understanding long-term changes in soil quality.

In this study, based on high-throughput sequencing technology, we depicted the distribution patterns, successional patterns, assembly mechanisms, and ecological networks of soil bacterial and fungal communities in *L. barbarum* fields with different stand ages, and we explored the relationship between tree age, soil physicochemical properties, microbial community diversity, and network complexity. It was hypothesized that i) the richest soil microbial communities are distributed in mid-aged stands, which exhibit the highest crop productivity compared with young and old stands; ii) the assembly processes of the soil microbial community and their ecological networks are influenced by increasing stand ages; and iii) the diversity of soil bacterial and fungal communities influences changes in their network complexity. The results of this study could provide guidance for the long-term management of *L. barbarum* fields.

## 2 Materials and methods

### 2.1 Study site and soil sample

The study site is located in Zhongning County (105°26′–106°07′E, 36°49′–37°50′N), Zhongwei City, Ningxia Hui Autonomous Region, China. Zhongning is renowned for having the best fruit quality and the largest production area of *L. barbarum* in Ningxia; the annual average altitude, annual precipitation, and mean temperature at the study site are 1,370 m, 244 mm, and 10.5°C, respectively (Wang et al., 2019). The major soil type is Anthrosols, and the soil texture is sandy loam.

*L. barbarum* field plots of four stand ages (3, 6, 10, and 15 years) were selected. Fertilizers, mostly Stanley compound fertilizer (N–P_2_O_5_–K_2_O), were applied twice a year at an average rate of 6,000–6,500 kg/hm^2^. In June 2022, six *L. barbarum* plants of similar growth in each plot were selected, and soil was collected from within its crown width (i.e., the root zone of the *L. barbarum*) after removing litter such as dead leaves from the surface soil. Each sample was separated into two parts. The first part of the sample was used to determine soil physicochemical properties, while the second part was stored at −80°C until it was used for the analysis of microbial communities.

### 2.2 Soil property analyses

The soil physicochemical parameters (**Table 1**) were obtained using the standardized method of Lu (2000). The details are as follows: soil pH and total salt (TS) content were measured in suspensions at a soil/water ratio of 1:5 (w/v) using a pH meter (PHS-3C; INESA Scientific Instrument Co., Nanjing, China) and a salinity meter (VZ8371BZ; AZ Instrument Corp., Taipei, China). Soil water content (SWC) was determined using the drying method, i.e., the ratio of the difference in weight of the soil sample before and after drying by 105℃ to the weight of the soil sample before drying. Additionally, ammonium–nitrogen (NH_4_^+^–N) and nitrate–nitrogen (NO_3_^−^–N) were quantified using the distillation method and ultraviolet spectrophotometry, respectively. The determination of available phosphorus (AP) and potassium (AK) contents was based on the Olsen method and ammonium acetate extraction–flame photometry, respectively. Alkali-hydrolyzable nitrogen (AN) and organic matter (OM) were analyzed using the alkaline diffusion method and the potassium dichromate oxidation–external heating method, respectively.

**Table 1:** Soil physicochemical properties in wolfberry orchards of different ages.

Stand age	3 years	6 years	10 years	15 years
TS (g/kg)	0.63 ± 0.09a	0.77 ± 0.21a	0.76 ± 0.22a	1.25 ± 0.29b
pH	8.18 ± 0.12a	8.32 ± 0.18a	8.21 ± 0.07a	8.05 ± 0.16a
SWC (%)	14.77 ± 1.32a	14.51 ± 1.92a	11.90 ± 3.92a	13.38 ± 3.04a
OM (g/kg)	43.99 ± 10.88a	22.07 ± 3.66b	16.88 ± 8.68b	51.41 ± 9.75a
AN (mg/kg)	74.84 ± 8.48b	63.33 ± 8.70ab	68.57 ± 14.90ab	55.16 ± 7.79a
NH_4_^+^–N (mg/kg)	2.41 ± 0.74a	2.51 ± 2.25a	2.72 ± 1.49a	1.57 ± 0.80a
NO_3_^−^–N (mg/kg)	14.99 ± 3.66a	25.40 ± 9.90b	23.53 ± 4.77ab	28.34 ± 7.57b
AP (mg/kg)	79.78 ± 11.82ab	71.86 ± 12.93a	105.17 ± 15.38b	88.58 ± 29.41ab
AK (mg/kg)	359.00 ± 50.61a	372.33 ± 29.00a	330.00 ± 31.52a	396.22 ± 66.52a

Different letters represent significant differences (*p*<0.05) between soil physicochemical indicators for different tree ages. The below is same.

### 2.3 Soil DNA extraction and sequencing

According to the manufacturer’s instructions, total genomic DNA was extracted from 0.5-g soil samples using a FastDNA Spin Kit for Soil (MP Biomedicals, Santa Ana, CA, USA). The V4 variable region of the bacterial 16S ribosomal RNA genes was amplified by PCR using the primer pair of 515F (5′-GTGCCAGCMGCCGCGGTAA-3′) and 806R (5′-GGACTACNNGGGTATCTAAT-3′) (Ding et al., 2015). For fungi, the internal transcribed spacer region 1 (ITS1) was amplified by PCR using the primer pair of ITS5-1737F (5′-GGAAGTAAAAGTCGTAACAAGG-3′) and ITS2-2043R (5′-GCTGCGTTCTTCATCGATGC-3′) (Lu et al., 2013). The 30 μL PCR reactions contained 2× Phusion Master Mix (New England Biolabs, Ipswich, MA, USA), 1 μM of each primer, 10 μL template DNA (5–10 ng), and ultraclean water to make up the volume. PCR conditions were as follows: one cycle at 98°C for 1 min, followed by 30 cycles at 98°C for 10 s, 50°C for 30 s, and 72°C for 30 s, and a final extension step at 72°C for 5 min. All PCR products were checked using 2% agarose gel electrophoresis, and the target bands of 200–300 bp were recovered using a DNA Purification Kit (Tiangen, Beijing, China).

The concentration of purified PCR products was measured using NanoDrop 1000 (NanoDrop Technologies, Wilmington, DE, USA). The samples were equally mixed according to their concentrations to construct libraries. Sequencing was accomplished on an Illumina NovaSeq 6000 platform (https://www.novogene.com/). Furthermore, the raw reads were spliced and filtered to obtain clean data. Then, the sequences were identified and distinguished based on the barcode sequences to obtain valid data. The raw sequences of the microorganisms from this study have been submitted to the Sequence Read Archive (SRA) of the National Center for Biotechnology Information (NCBI), and the accession numbers for bacteria and fungi are PRJNA1215385 and PRJNA1215412, respectively.

### 2.4 Assembly of microbial communities

Neutral community models (NCMs), used to quantify the importance of stochastic processes in the assembly of bacterial and fungal communities, were carried out using the “hmisc”, “minpack.lm”, and “stats4” packages. Furthermore, a 50% Modified Stochasticity Ratio (MST) was used to assess the order of importance of deterministic and stochastic processes in microbial community assembly. Based on the Levins model, the habitat niche width of bacteria and fungi, obtained from the “spaa” package, were used to further explain the mechanisms by which microbial community assembly occurred (Fang et al., 2023).

### 2.5 Construction and analysis of microbial ecological networks

Based on the Gephi software (version 0.9.7), four ecological networks of bacteria and fungi were constructed across various stand ages of *L. barbarum*. In the construction process, Operational Taxonomic Units (OTUs) that satisfied both an average relative abundance of less than 0.1% and a representation in less than 1/3 of the total sample size were removed in order to control the complexity of the network and improve its accuracy. Then, using the “igraph” and “psych” packages, Spearman’s correlation of the screened OTUs across various stand ages of *L. barbarum* was calculated. Only strong (correlation coefficient |r| > 0.70) and significant relationships (significance *p* < 0.01) were retained in the networks. The stability of the microbial networks was assessed by calculating their robustness, which evaluates the reliability of the remaining nodes after randomly removing 50% of the nodes in the network. The above analyses refer to the description in Zhang et al. (2024a). In addition, the subgraph function in the “igraph” package was used to extract the subnetworks from each network, and then the average degree of each subnetwork was computed, which serves as a measure of the network’s complexity (Zhang et al., 2024b).

### 2.6 Data statistical analysis

R language (version 4.2.3) and SPSS (version 22.0) were used to process and statistically analyze the data. For the bacterial and fungal community, the “Vegan” package was utilized to analyze their α-diversity metrics (Shannon and Chao1 indices), conduct the β-diversity, and perform the Adonis test (He et al., 2024a). The β-diversity of microbial communities, which reflects dissimilarities in microbial community composition across different treatments, was visualized using principal coordinates analysis (PCoA). The β-diversity decomposition was further employed to evaluate the sources of differences in microbial community composition, i.e., turnover or nestedness, based on the “betapart” package (Wu et al., 2024b). The linear discriminant analysis effect size (LEfSe) method was implemented to identify differences in bacterial and fungal communities at the top 15 genera across various stand ages using the “microeco” package. The thresholds for the Linear Discriminant Analysis (LDA) score and the p-value were set at 2 and 0.1, respectively (Zhang et al., 2024c).

The partial least squares path modeling (PLS-PM) was used to identify the relationships between observed and latent variables, specifically to assess the effects of stand age, physicochemical properties, and microbial communities on network complexity using the “plspm” package. In this model, forward selection was conducted to obtain the following best revealed variables: stand age, physicochemical properties (including OM, AN, NH_4_^+^–N, NO_3_^−^–N, AP, and AK), bacterial or fungal community (represented by the Shannon index), and network complexity (measured by edges and average degree). Stand age was classified as an exogenous variable, physicochemical properties and microbial communities were classified as endogenous variables, and network stability was classified as a response variable. It is worth noting that variables with low loadings (loading < 0.7) were excluded, and the predictive performance of the model was evaluated using the goodness of fit (GOF) index.

## 3 Results

### 3.1 Diversity of soil bacterial and fungal communities

**Figure 1:**
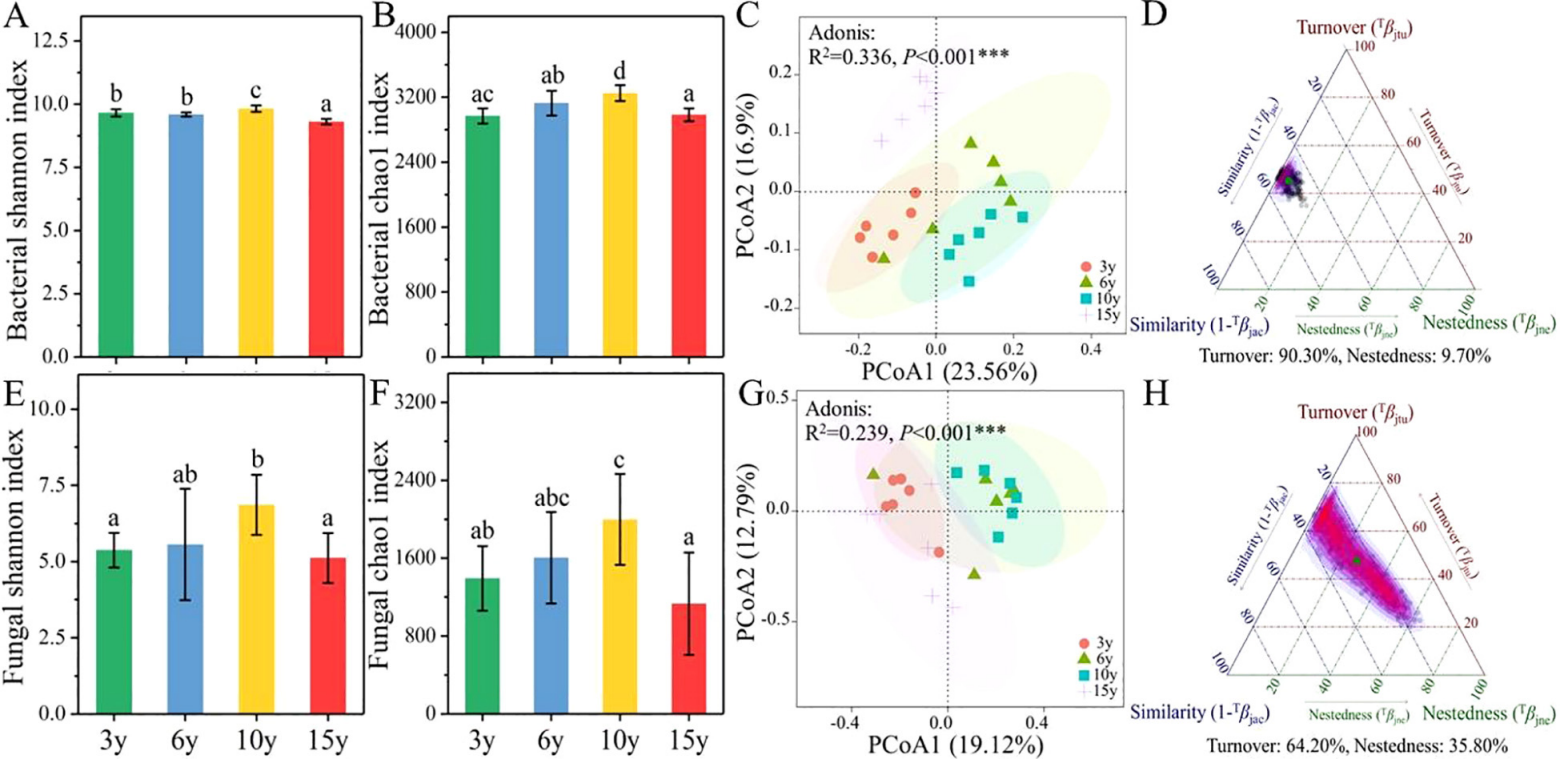
The α-diversity index of bacterial **(A, B)** and fungal communities **(E, F)** in the root zone soils of *Lycium barbarum* of different ages. The PCoA analysis of bacterial **(C)** and fungal **(G)** communities. The β-diversity decomposition indices of bacterial **(D)** and fungal **(H)** communities based on Bray–Curtis distance. PCoA, principal coordinates analysis. *** indicate significant differences between groups at significance levels of 0.001. The below is same.

The effect of long-term continuous cultivation on the diversity and composition of soil microbial communities in the root zone of *L. barbarum* is evident. The α-diversity of bacterial (**Figures 1A, B**) and fungal (**Figures 1E, F**) communities, characterized by the Shannon and Chao1 indices, exhibited a tendency to increase and then decrease with increasing tree age. Specifically, soil bacterial and fungal communities in the root zone of 10-year-old *L. barbarum* possessed the highest diversity and abundance compared to those of other tree ages. The results of PCoAs based on the Bray–Curtis distance revealed that the β-diversity of soil bacterial (Adonis: R^2^ = 0.336, *p* < 0.001) and fungal communities (Adonis: R^2^ = 0.239, *p* < 0.001) was significantly differentiated between tree ages, confirming the significant effect of stand age on the composition of soil bacterial and fungal communities in the root zone of *L. barbarum* (**Figures 1C, G**). Meanwhile, β-diversity decomposition indices for soil bacterial and fungal communities in the root zone of *L. barbarum* of different ages were calculated to ascertain the primary reasons for the differences in microbial communities (**Figures 1D, H**). The results indicated that differences in soil bacterial and fungal communities among tree ages were primarily attributed to species replacement (bacteria, 90.30%; fungi, 64.20%).

### 3.2 Composition of soil bacterial and fungal communities

**Figure 2:**
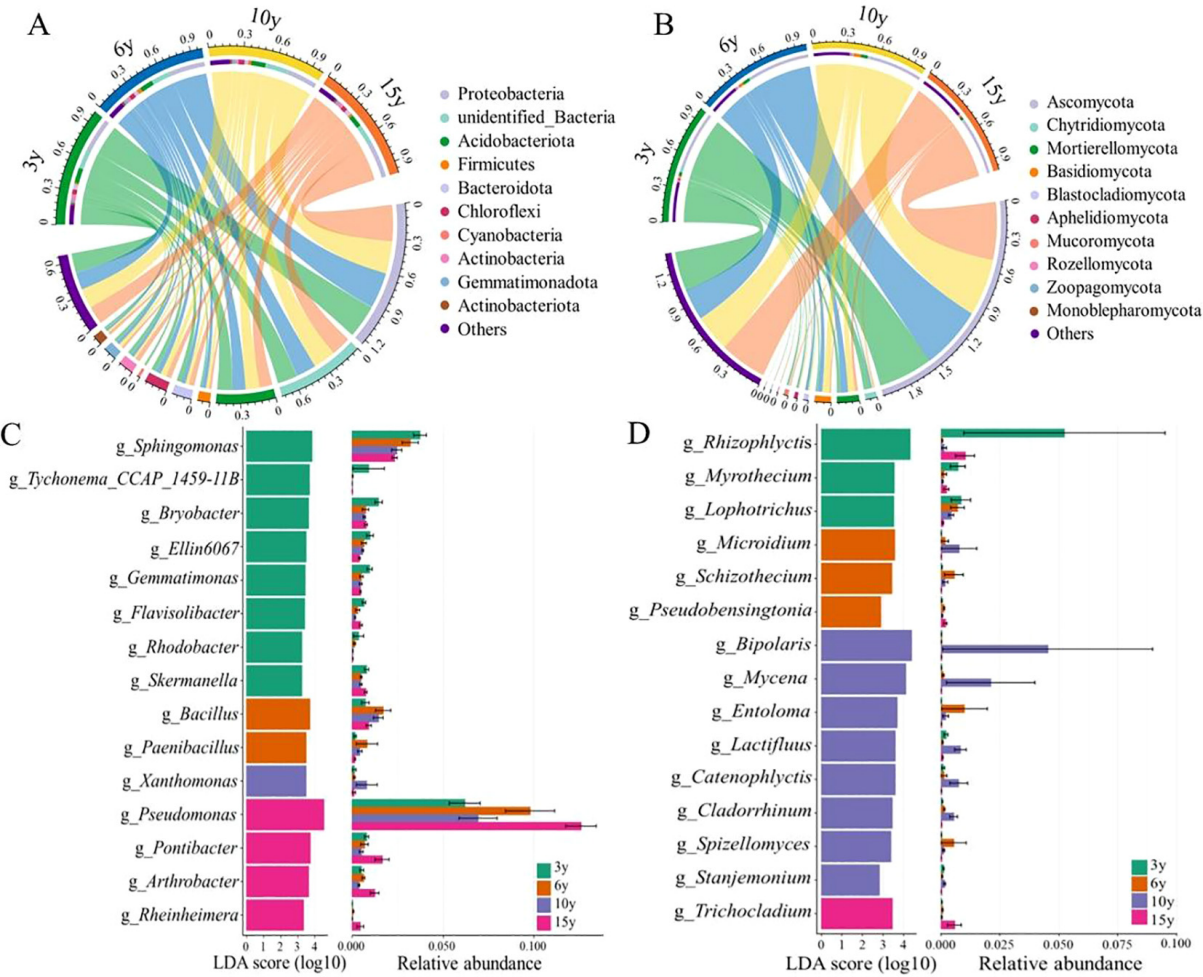
Composition of soil bacterial **(A)** and fungal **(B)** phyla in the root zone of four tree-aged wolfberry orchards. LEfSe analysis of soil bacterial **(C)** and fungal **(D)** genera in the root zone of four tree-aged wolfberry orchards. LEfSe, linear discriminant analysis effect size.

The composition of soil bacterial and fungal communities in the root zone of *L. barbarum* of different ages exhibited dramatic variations at the phylum level (**Figures 2A, B**). Proteobacteria (28.77%–32.81%) and Ascomycota (49.72%–55.82%) were predominantly dominant among the bacterial and fungal phyla, respectively. As the stand age of *L. barbarum* increased, the relative abundance of the bacterial phylum Actinobacteria showed an increasing trend, whereas that of Gemmatimonadota exhibited a decreasing trend. Compared to that of bacteria, the abundance of fungal phyla varied more markedly in the root zone soils of *L. barbarum* of different ages; for example, 15-year-old *L. barbarum* root zone soils had lower abundances of Mortierellomycota, Basidiomycota, Blastocladiomycota, Aphelidiomycota, Rozellomycota, and Zoopagomycota.

LEfSe analyses, which assessed differences in the abundance of microbial taxa at the genus level in the soil of the root zone of *L. barbarum* of different ages, provided results that showed the most representative bacterial and fungal information in the soil of each stand age (**Figures 2C, D**) . For bacteria, the genera *Sphingomonas*, *Tychonema_CCAP_1459-11B*, *Bryobacter*, *Ellin6067*, *Gemmatimonas*, *Flavisolibacter*, *Rhodobacter*, and *Skermanella* exhibited superior relative abundance in the root zone soil of 3-year-old *L. barbarum* plants compared to the other tree age groups. In contrast, the genera *Pseudomonas*, *Pontibacter*, *Arthrobacter*, and *Rheinheimera* were more prevalent in 15-year-old *L. barbarum* than in 3-, 6-, and 10-year-old *L. barbarum*. Half of these genera belong to the phylum Proteobacteria, while the others belong to Firmicutes, Bacteroidota, Actinobacteria, Acidobacteriota, Cyanobacteria, and Gemmatimonadetes. Similarly, for fungi, there were differences in the dominant genera enriched in the root zone soil of *L. barbarum* of different stand ages, which belonged to the phyla Ascomycota, Basidiomycota, Blastocladiomycota, and Chytridiomycota. In particular, more genus-level biomarkers were found in 10-year-old trees, such as *Bipolaris*, *Mycena*, *Entoloma*, *Lactifluus*, *Catenophlyctis*, *Cladorrhinum*, *Spizellomyces*, and *Stanjemonium*.

### 3.3 Microbial community assembly processes and ecological niche width

**Figure 3:**
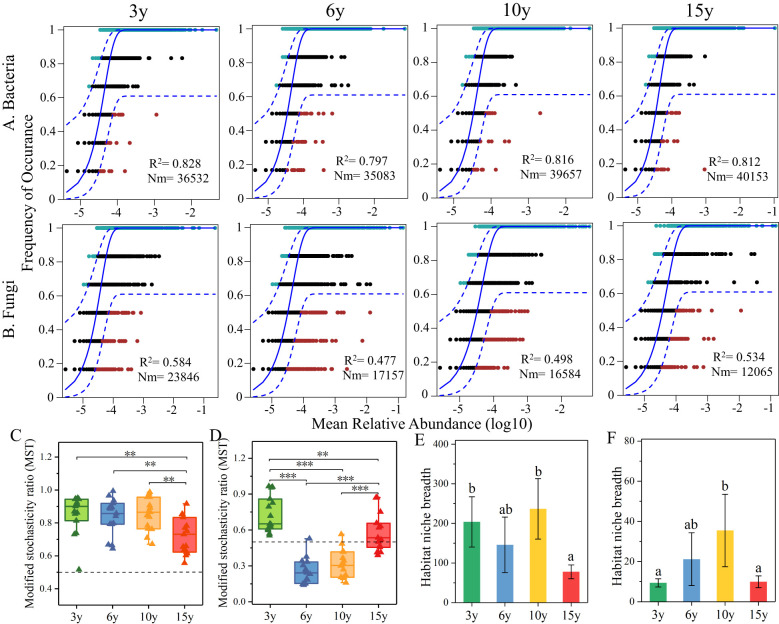
The weight of stochastic processes in bacterial **(A)** and fungal **(B)** community assembly across various stand ages of wolfberry orchards, predicted using NCMs. The blue solid and dashed lines represent the best fit to the neutral model and the 95% confidence intervals of the model predictions, respectively. R^2^ represents the overall fit to the neutral model. Nm indicates the assessment of within-community estimated migration. MST was used for assessing the ratios of stochastic and deterministic processes in the assembly of bacterial **(C)** and fungal **(D)** communities across various stand ages. Ecological niche widths of bacterial **(E)** and fungal **(F)** communities. NCMs, neutral community models. ** and *** indicate significant differences between groups at significance levels of 0.01 and 0.001, respectively. The below is same.

In this study, a neutral model and a modified stochastic ratio null model were utilized to assess the assembly processes of soil bacterial (**Figure 3A**) and fungal (**Figure 3B**) communities in the root zone of *L. barbarum* of different ages. The results of the neutral model revealed the dominance of stochastic processes in the construction of bacterial communities across the plantation chronosequence, with stochastic processes explaining 79.7%–82.8% of the variance. Bacterial taxa exhibited higher Nm values at 10 (Nm = 39,657) and 15 years of age (Nm = 40,153) compared to those at 3 (Nm = 36,532) and 6 years (Nm = 35,083), suggesting that species dispersal within bacterial communities is higher at 10 and 15 years of age than at 3 and 6 years. The assembly process of the fungal community seemed to fluctuate between different tree ages, and the contribution of stochastic processes accounted for 47.70%–58.40% of the variance. The Nm value of the fungal community exhibited an overall decreasing tendency with increasing tree age, indicating that the species dispersal within the fungal community was limited by the stand age to a certain extent. MST analyses further confirmed the significance of stochastic processes in the assembly of bacterial communities (**Figure 3C**). Conversely, the assembly of fungal communities (**Figure 3D**) exhibited fluctuations due to stand age, which were driven by a combination of stochastic and deterministic processes. Additionally, the ecological widths of bacteria (**Figure 3E**) and fungi (**Figure 3F**) displayed an overall trend of increasing and then decreasing. Specifically, they exhibited the highest and lowest ecological niche widths at 10 and 15 years of age, respectively. It is noteworthy that bacteria demonstrated a broader range of ecological widths compared to fungi.

### 3.4 Molecular ecological networks of soil bacterial and fungal communities

**Figure 4:**
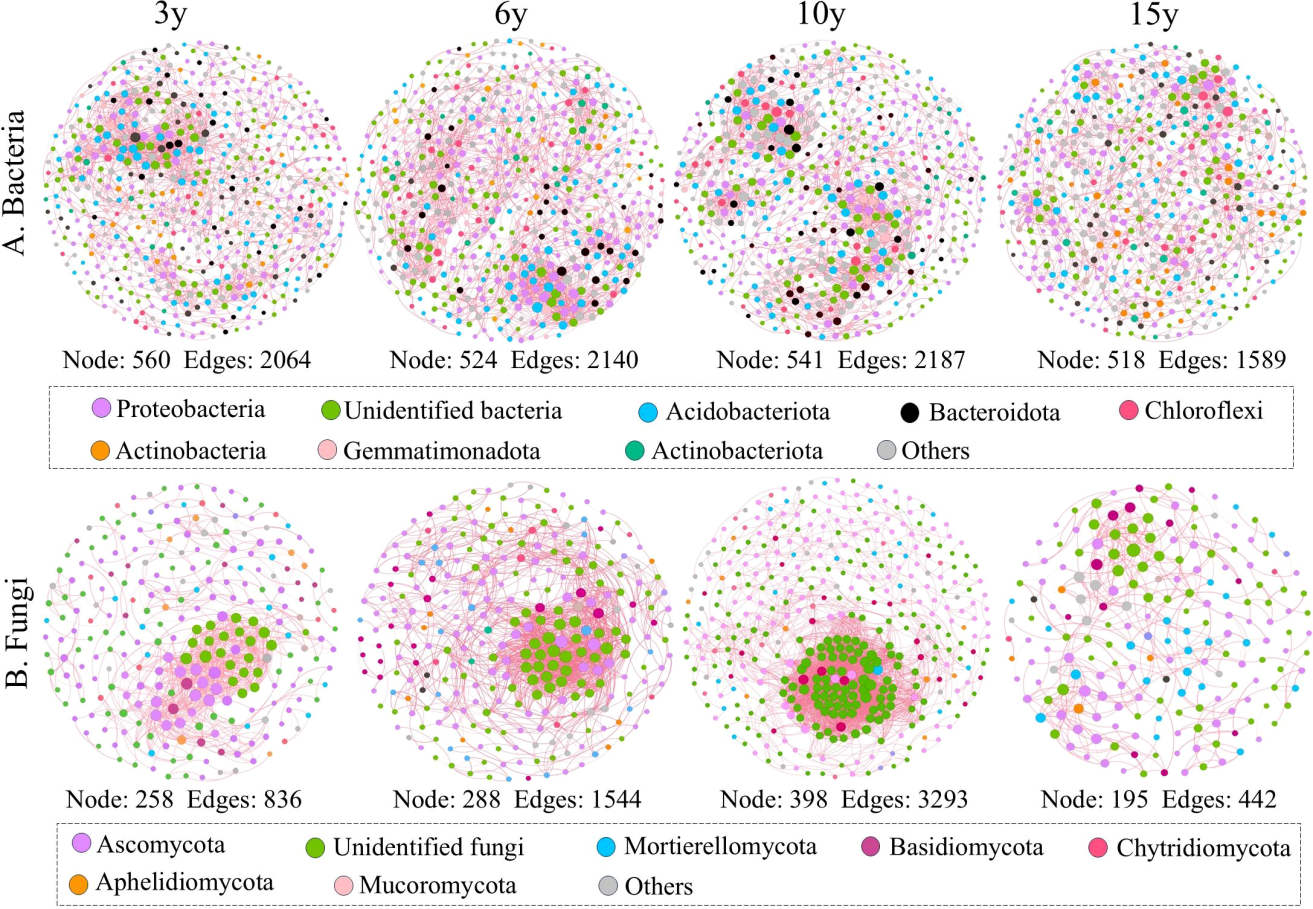
Ecological networks of bacterial **(A)** and fungal **(B)** communities in the root zone soil of *Lycium barbarum* orchards at different ages (3, 6, 10, and 15 years). Nodes in the network are colored according to different bacterial and fungal phyla. Positive and negative correlations between different nodes are shown by the pink and blue lines, respectively.

We constructed ecological networks for bacterial and fungal communities and calculated their topological parameters to assess species interactions in soils from the root zone of four wolfberry plants of different ages (**Figure 4**; **Table 2**). The results revealed that the complexity of the bacterial and fungal networks exhibited an increasing and then decreasing trend as stand age increased. For bacteria, the 15-year-old network was clearly distinct from the other age networks, with the lowest complexity, having up to 27.34% fewer connections and a 24.89% lower average degree than the other age networks. For fungi, the network complexity followed the order of 15 years < 3 years < 6 years < 10 years. The 10-year-old fungal network was the most complex, primarily because it had the highest number of links (3,293) and average degree (16.548) compared to the 3-, 9-, and 15-old networks (edges, 836, 1544, and 442, respectively; average degree, 6.481, 10.722, and 4.533, respectively). Interestingly, the ecological networks of both bacteria and fungi were dominated by positive associations. Furthermore, we quantitatively assessed the robustness of the microbial ecological network by randomly removing 50% of the nodes, which served as an indicator of the network stability. The results confirmed that the stability of both the 15-year-old bacterial and fungal networks was the lowest (bacteria, 0.279; fungi, 0.276). Notably, the highest network modularity values were observed in the 15-year-old bacterial and fungal networks, but not in those of the other stand ages. In addition, further analysis revealed that the bacterial phyla Proteobacteria, Unidentified bacteria, and Acidobacteriota, as well as the fungal phyla Ascomycota and Unidentified fungi, were the key nodes for maintaining the respective bacterial and fungal networks.

**Table 2:** Topological parameters of bacterial and fungal networks in the root zone soil of *Lycium barbarum* of different ages.

	Bacteria				Fungi			
	3 years	6 years	10 years	15 years	3 years	6 years	10 years	15 years
Nodes	560	524	541	518	258	288	398	195
Edges	2,064	2,140	2,187	1,589	836	1,544	3,293	442
Average degree	7.371	8.168	8.085	6.135	6.481	10.722	16.548	4.533
Network diameter	23	19	23	17	17	21	21	19
Density	0.013	0.016	0.015	0.012	0.025	0.037	0.042	0.023
Average path length	7.837	6.835	7.193	6.968	5.703	6.135	6.684	5.78
Clustering coefficient	0.376	0.345	0.378	0.387	0.351	0.354	0.378	0.342
Modularity degree	0.731	0.753	0.762	0.833	0.58	0.521	0.378	0.743
Positive (%)	99.47	99.81	99.77	99	100	99.81	99.97	100
Robustness	0.308	0.294	0.297	0.279	0.290	0.365	0.316	0.276

### 3.5 Relationships among stand age, diversity of the microbial community, and network complexity

**Figure 5:**
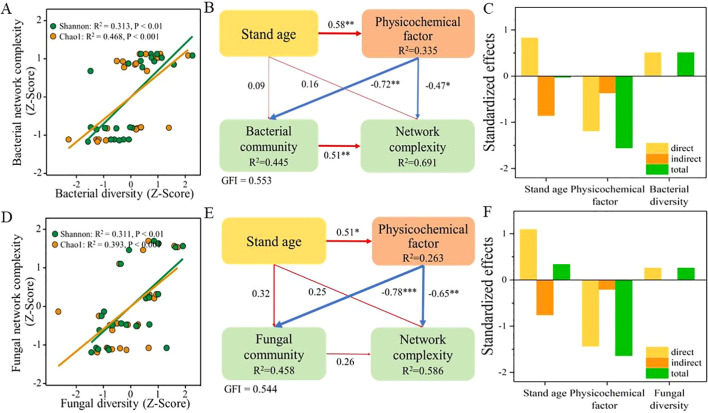
Correlation analysis between microbial community diversity and ecological network complexity (**A**, bacteria; **D**, fungi). PLS analysis reveals direct and indirect effects of tree age, physicochemical properties, and microbial communities on network complexity (**B**, bacteria; **E**, fungi). Standardized effects of various factors on the complexity of bacterial **(C)** and fungal **(F)** networks. PLS, partial least squares. * and ** indicate significant differences between groups at significance levels of 0.05 and 0.01, respectively. The below is same.

To determine the relationship between the diversity of soil microbial communities and their network complexity, correlation analyses were performed (**Figures 5A, D**). The results revealed a significant positive correlation between the Shannon (bacteria, *p* < 0.01; fungi, *p* < 0.01) and Chao1 (bacteria, *p* < 0.001; fungi, *p* < 0.001) indices of microbial communities and the complexity of their respective networks. This confirmed the close relationship between the diversity of soil microbial communities in the root zone of *L. barbarum* of different ages and the complexity of their networks. Further, PLS-PM analyses were conducted to assess the effects of stand age, physicochemical properties, and microbial community diversity on the complexity of the microbial network (**Figures 5B, E**). The results indicated that stand age had a direct and strong significant effect (*p* < 0.05) on the physicochemical properties of the soil, but not on the diversity of bacterial and fungal communities. However, soil physicochemical properties exhibited significant and direct adverse effects on the diversity of both bacterial (path coefficient, −0.72, *p* < 0.01) and fungal (path coefficient, −0.78, *p* < 0.001) communities. Furthermore, soil physicochemical properties also had a significant negative regulatory effect on the complexity of both bacterial (path coefficient, −0.47, *p* < 0.05) and fungal (path coefficient, −0.65, *p* < 0.01) networks. It is noteworthy that a significant and positive relationship persisted between the diversity of bacterial communities (path coefficient, 0.51, *p* < 0.01) and the complexity of their networks when considering the effects of stand age, physicochemical properties, and microbial community on network complexity. However, this was not the case for fungal communities (path coefficient, 0.26, *p* > 0.05). In summary, stand age can indirectly regulate the diversity of bacterial and fungal communities, as well as the complexity of their networks, by influencing soil physicochemical properties. Additionally, the diversity of bacterial communities, but not fungal communities, exhibited a strong and positive regulatory effect on their network complexity. The results of the standardized effects analysis (**Figures 5C, F**) revealed that soil physicochemical properties had a significant impact on the complexity of both bacterial and fungal communities, emerging as the strongest predictors of bacterial and fungal network complexity. Following closely were the stand age and diversity of bacterial and fungal communities, which served as the second and third most influential factors, respectively, emphasizing their notable impact on network complexity.

## 4 Discussion

### 4.1 The diversity and composition of soil microbial communities in the root zone of *L. barbarum* are affected by stand age

Long-term monoculture of *L. barbarum* can result in changes in the soil microbial community. Microorganisms, as crucial components of soil ecosystems, contribute to the decomposition of organic matter and nutrient cycling and play a pivotal role in the creation of healthy soil habitats (Liu et al., 2020a). The results of this study confirmed the highest α-diversity among bacterial and fungal communities in middle-aged *L. barbarum* stands and the lowest in old-aged stands, respectively, thus supporting our first hypothesis. This may be attributed to the availability of soil nutrients following continuous monoculture over an extended period, where the levels of soil nutrients (e.g., C, N, and K) dictate soil microbial growth and activity (Zhang et al., 2023b). Although mid-aged stands with vigorous growth may consume a large amount of soil nutrients, farmers often apply higher levels of fertilizers at this stage to ensure high crop yields. At the same time, during periods of vigorous plant growth, the incidence of plant apomixis also increases, which, to some extent, raises the level of soil nutrients and enhances the soil microenvironment (Gong et al., 2024). The abundance of soil nutrients is also beneficial for the propagation of microbial communities. Numerous studies have established a positive correlation between soil nutrient levels and the diversity of bacteria and fungi (Semenov et al., 2018). For example, OM, AK, and AP were found to be the main factors influencing the distribution of soil bacterial communities in the rhizosphere of cucumber in a study by Huang et al. (2023).

Consistent with previous findings, our results indicated that Proteobacteria and Ascomycetes were the most abundant phyla among soil bacterial and fungal communities, respectively, in the root zone of *L. barbarum*. This may be attributed to their strong adaptability and survivability, which allow them to be widely distributed in various types of agricultural soils (Song et al., 2024). Other bacterial and fungal taxa also exhibited quantitative advantages at different tree ages, particularly fungi, which are known for their role in decomposing soil organic matter (such as plant and animal residues, including dead leaves) (Barberán et al., 2014). For example, Mortierellomycota, serving as an indicator of soil organic matter and nutrient levels, decomposes soil organic compounds like sugars and is significantly enriched in root zone soils of mid-aged (6-year-old and 10-year-old) *L. barbarum* (Fu et al., 2020). In addition, LEfSe analyses screened for markers of bacterial and fungal genera across different tree ages, suggesting that some specific microbial taxa play their key roles only under certain specific conditions.

### 4.2 Soil microbial community assembly and network complexity were affected by the stand age of *L. barbarum*

Previous studies have shown that long-term continuous monoculture significantly impacts microbial community assembly processes, leading to alterations in the diversity and composition of soil microbial communities (Liu et al., 2020b). Our results indicated that, although stochastic processes predominantly governed the assembly of bacterial communities, the influence of stochastic processes in the soil bacterial community of old-aged *L. barbarum* was significantly lower than that observed in other tree ages. This reflects the influence of increasing tree age on the structural changes in microbial communities under long-term continuous cultivation conditions (Zhou et al., 2024). Compared to bacterial communities, stochastic processes play a notably less important role in fungal communities, emphasizing the significant impact of changes in the soil environment under long-term cultivation on fungal communities. Generally, the theory of “body size plasticity” suggests that larger organisms are more susceptible to external environmental factors than smaller organisms; hence, fungi, being typically larger than bacteria, are more susceptible to external environmental factors than bacteria (Farjalla et al., 2012). In terms of ecological niche width, fungi generally occupy a narrower niche than bacteria, implying that fungi have access to fewer resources than bacteria. This contributes to the dominance of stochastic processes (rather than deterministic processes) in bacterial community assembly, as bacteria can tolerate and exploit a wider range of environmental conditions (He et al., 2024a).

Analysis of microbial ecological networks offers valuable insights into the complexity and stability of microbial community assemblages in response to aging. It is generally accepted that complex and stable networks contribute to the potential for soil ecosystem services, which are crucial for maintaining sustainable soil ecosystems (Huo et al., 2023). Alterations in bacterial and fungal ecological networks underscore the impact on associations between bacterial and fungal species, aligning with our second hypothesis. A previous study by Na et al. (2021) also found that networks in younger stands had more nodes, edges, and higher average degrees than those in older stands, confirming the presence of negative feedbacks between plants and soil beyond a certain number of planting years. Differences in microbial ecological networks imply that the interactions between microbial taxa present in a given environment are different and to some extent represent the degree of connectedness between microbial taxa. Interestingly, a high proportion of positive associations exist in all these networks, implying that cooperation rather than competition dominated the process of assembling soil bacterial and fungal communities in the root zone of *L. barbarum*. Generally, positive associations symbolize positive interactions and symbiosis, which can enhance resistance to the external environment (Coyte et al., 2015).

### 4.3 Relationships between stand age, physicochemical property, microbial community, and network complexity

Based on PLS-PM analyses, we found that tree age primarily modulates microbial community diversity and network complexity by influencing physicochemical properties, thereby emphasizing the strong influence of soil nutrient content on soil bacterial and fungal communities. Similar conclusions were reached by Yi et al. (2023), who found that soil physicochemical properties are significantly and strongly influenced by region and play a crucial role in the succession of microbial communities. Soil physicochemical factors have been recognized as key determinants of microbial community diversity and composition (Zhou and Zhou, 2023). Furthermore, stand age may also influence the complexity of bacterial and fungal networks indirectly through physicochemical properties, suggesting that soil nutrient content may facilitate interactions and symbioses within soil bacterial and fungal communities (Wu et al., 2024a).

Regarding symbiotic networks of bacteria and fungi, previous studies have primarily focused on the effects of abiotic factors, such as tree age and physicochemical properties, on their complexity and stability (Wang et al., 2023; Niu et al., 2024). For instance, He et al. (2024a) discovered that salinity gradients were dominant in shaping bacterial symbiotic networks, while Yang et al. (2023) found that elevation gradients had a similar influence on fungal symbiotic networks. Our results revealed that in addition to abiotic factors, biotic factors—particularly the diversity of the bacterial community—exert an extremely significant and strong influence on the complexity of its network. This implies that the presence or absence of specific bacterial community members not only affects their interactions but also plays a pivotal role in the formation of the entire network (Li et al., 2020; Yu et al., 2024). Although the diversity of fungal communities exhibited significant differences across tree ages, there was no notable effect on the complexity of their networks. This may be attributed to the overlapping ecological niches and functional redundancy within the fungal communities (Zeng et al., 2023). In conclusion, higher microbial diversity and network complexity generally indicate more stable ecological processes, which are crucial for the stability and sustainability of agroecosystems.

## 5 Conclusion

This study provides a comprehensive understanding of the effects of long-term monoculture of *L. barbarum* on soil microbial community diversity, composition, assembly, and network properties. The results indicated that tree age exerts a significant effect on bacterial and fungal communities. The diversity of these communities increased with tree age but decreased in older *L. barbarum* stands, emphasizing the selective pressure exerted by tree age on microbial populations. The composition of bacterial and fungal communities varied with tree age, with some phyla and genera showing dominance in soils of different ages. For instance, LEfSe analysis identified several bacterial and fungal genera as biomarkers that differentiate tree ages, emphasizing their role in the root zone soils of *L. barbarum* at various ages. In addition, the complexity and stability of bacterial and fungal networks exhibited a tendency to increase and then decrease with stand age, highlighting the disruption caused by old-aged *L. barbarum* cultivation to the linkages within the soil microbial community. While the assembly of bacterial communities was consistently dominated by stochastic processes as tree age increased, the assembly of fungal communities appeared to be more variable, underscoring the constraints imposed by specific environments of different tree ages on fungal community assembly. Tree age emerged as a pivotal factor influencing microbial community diversity and network complexity, with these effects primarily mediated by physicochemical properties. The findings of this study imply that nutrient management during *L. barbarum* cultivation should be enhanced, and old, low-productivity *L. barbarum* plants should be replaced in a timely manner. This is crucial for maintaining the health of soil ecosystems in *L. barbarum* gardens.

## Data Availability

The original contributions presented in the study are publicly available. This data can be found here: NCBI BioProject, accession numbers PRJNA1215385 (for bacteria) and PRJNA1215412 (for fungi).
